# Bilateral carpal tunnel syndrome revealing an acromegaly: a case report

**DOI:** 10.11604/pamj.2023.44.186.39745

**Published:** 2023-04-20

**Authors:** Fatima Toulali, Hajar Srifi, Dounia Talbi, Ahmed Anas Guerboub

**Affiliations:** 1 Endocrinology and Metabolic Diseases Department at the Mohammed V Military Hospital in Rabat, Rabat, Morocco

**Keywords:** Carpal tunnel syndrome, acromegaly, case report

## Abstract

Carpal tunnel syndrome (CTS) is the most common neuropathy in acromegalic patients and is often the initial complaint. However, the diagnosis of acromegaly is often made years after the diagnosis of CTS. In our case report, we describe the case of a patient in whom acromegaly was discovered after presenting bilateral carpal tunnel syndrome, without having acrofacial signs. Increased awareness of signs of acromegaly in patients with CTS might help to shorten the diagnostic delay in acromegaly.

## Introduction

Described for the first time in these terms by Pierre Marie, in 1886, acromegaly is linked to the hypersecretion of growth hormone by a pituitary adenoma. Its clinical manifestations are very large, and many of acromegalic patients suffer from different complications before the doctors think about acromegaly. Carpal tunnel syndrome is among the first signs that appear in acromegaly. Its bilateral character must make us think of the diagnosis [1]. In this article, we describe the case of a patient in whom the diagnosis of acromegaly was made only after she presented bilateral carpal tunnel syndrome and well before the development of the acrofacial signs.

## Patient and observation

**Patient information:** we report the case of a 56-year-old patient, with a history of total thyroidectomy for goiter.

**Figure 1:**
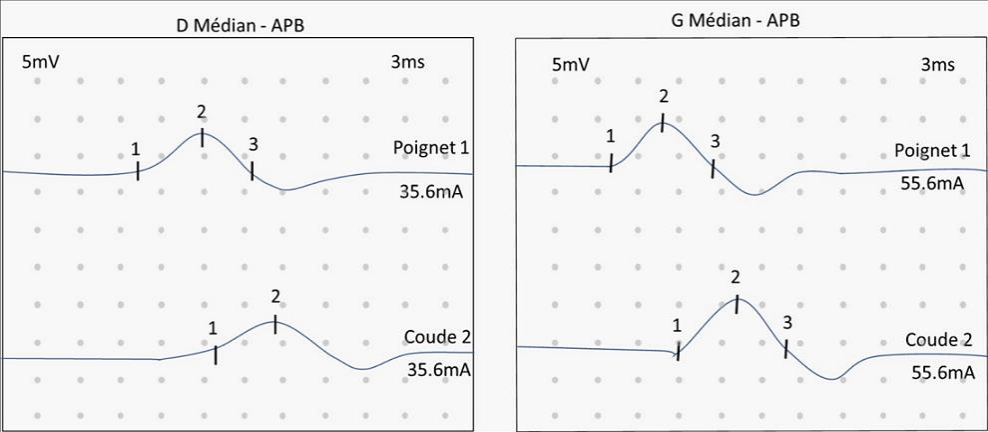
electrophysiological study showing bilateral delay of the distal motor latencies (DML) of the median nerves with reduction in the amplitudes of their motor and sensory responses and marked reduction in their sensory conduction velocities (SCV)

**Timeline:** the history of her disease goes back four years with the installation of pain in both hands with numbness and tingling. The pain affected the palmar surface of the thumb, index, and middle finger. An electrophysiological study was realized. It showed a bilateral delay of the distal motor latencies (DML) (35.4 ms) of the median nerves, a reduction in the amplitudes of their motor and sensory responses, and a marked reduction in their sensory conduction velocities (SCV) (Figure 1). It concluded with bilateral canal tunnel syndrome. Decompression surgery was realized two months after. Given the bilateral nature of the carpal tunnel syndrome and the history of thyroidectomy for goiter, the endocrinologist thought about acromegaly.

**Figure 2:**
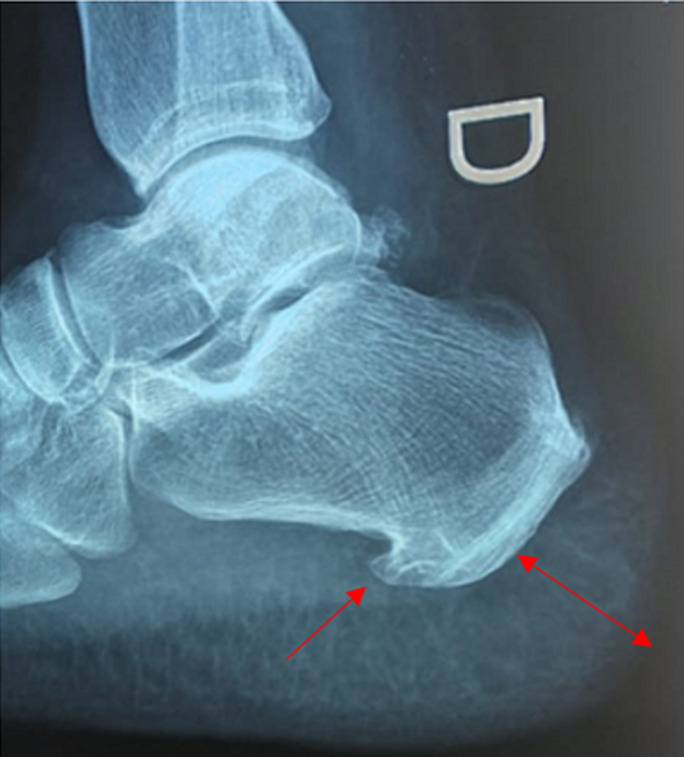
X-ray of foot showing the plantar pad and the osteophyte

**Figure 3:**
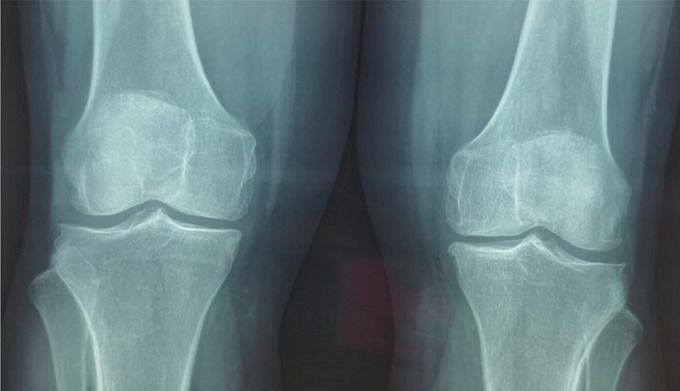
X-ray of knees showing widening of the joint space and bony hypertrophy of the condylar shells

**Figure 4:**
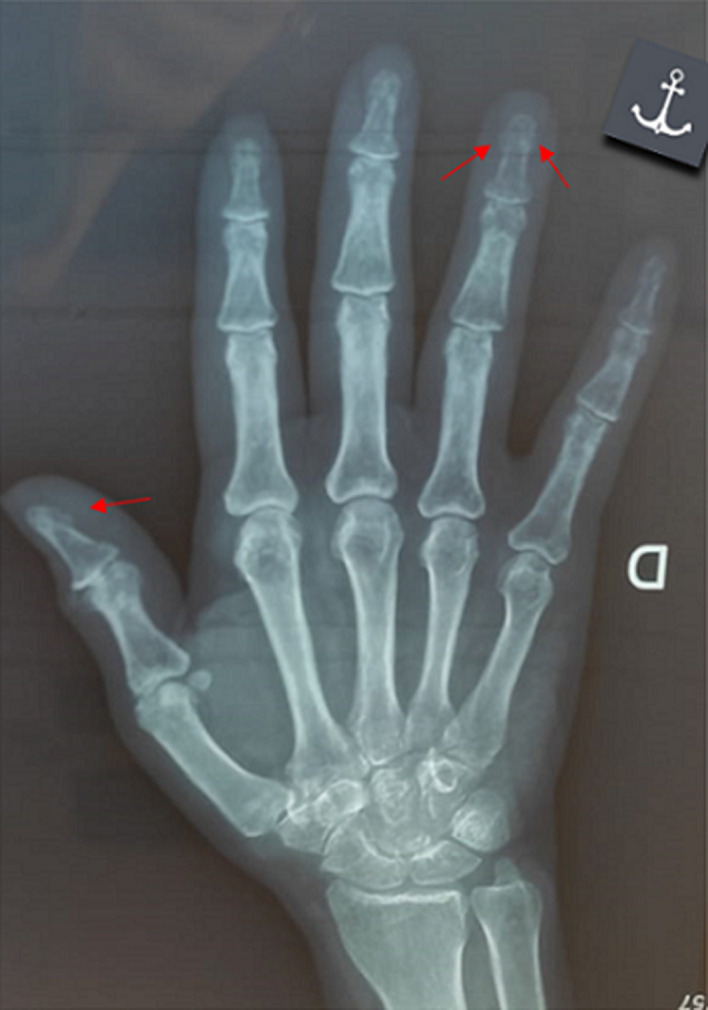
X-ray of the hand showing a sea anchor osteophytosis

**Clinical findings:** the careful questioning of the patient objectified a change in shoe size from 39 to 41, as well as a change in the voice becoming hoarse. The clinical examination revealed a moderate widening of the feet and hands as well as pain on the mobilization of the knees. The foot X-ray showed a footpad measured at 27 mm (>24 mm) and osteophytes (Figure 2). X-ray of the knee showed a widening of the space and bony hypertrophy of the condylar shells (Figure 3). And that of the hands showed a marine anchor osteophytosis at the level of the 3^rd^ phalanx (Figure 4). In view of these direct and indirect elements, acromegaly was suspected.

**Diagnostic assessment:** the diagnosis of acromegaly was confirmed biologically by high levels of GH (growth hormone), IGF1 (insuline-like growth factor-1), and by the absence of GH suppression following the oral glucose tolerance test. It was confirmed biologically by high levels of GH, IGF1, and by the absence of GH suppression following the oral glucose tolerance test. Growth hormone and IGF1 values were respectively: 5.13 mIU/l (Normal < 1.2 mIU/l), and 374 ng/ml (normal: 48-241). Morphologically, an MRI with dynamic imaging of the pituitary gland was performed. It showed a pituitary microadenoma of 5 x 3 mm, located on the right, in intimate contact with the homolateral carotid artery.

**Therapeutic intervention:** concerning CTS, decompression surgery was realized before the diagnosis of acromegaly. The pituitary adenoma was scheduled for surgery by transsphenoidal approach.

**Follow-up and outcomes:** the patient reports improvement in CTS symptoms after median nerve decompression surgery. An evaluation after pituitary adenoma surgery was planned.

**Patient perspective:** the patient was satisfied after the CTS decompression surgery, and she gave her consent for the pituitary adenoma surgery.

**Informed consent:** written informed consent was obtained from the patient.

## Discussion

Carpal tunnel syndrome (CTS) is a peripheral compression neuropathy. Its prevalence in the general population is about 3-5% [2]. In acromegaly, CTS is a common complication. It occurs in 20-64% of patients [3] and it is frequently bilateral [1]. In acromegaly, the mechanism of CTS involves compression of the median nerve by edematous synovial tissues. The latter is related to the excessive secretion of growth hormone which causes an increase in sodium and water retention in the extracellular fluid [4]. Another hypothesis about the mechanism of CTS in acromegaly seems to be nerve conduction impairment after nerve enlargement at the carpal tunnel. In acromegalic patients, IGF-1 can directly influence nerve tissue and causes enlargement of the median nerve [5].

The patient usually complains of a sensation of swelling or tension in the hand or forearm. Sometimes the symptomatology can reach a lack of sensitivity, typical nocturnal paraesthetic brachialgia. Motor disorders can also be observed in the hand muscles supplied by the median nerve [6]. One of the important studies that have investigated the relationship between acromegaly and CTS is the Swedish nationwide study realized in 2021. It included 599 patients. Its results showed that CTS surgery was performed before the diagnosis of acromegaly with an incidence 6-fold higher than the general population [6]. They found that the majority of acromegalic patients were diagnosed with CTS prior to acromegaly [6].

In our case, the presence of bilateral carpal tunnel syndrome in our patient suggested acromegaly, even in the absence of acrofacial syndrome. Biological assays of GH and IGF-1 confirmed the diagnosis of the latter. The magnetic resonance imaging showed the presence of a pituitary adenoma. Regarding the delay between the diagnosis of CTS and acromegaly, studies based on questionnaires have shown that acromegalic patients report symptoms of CTS on average 6 years before the diagnosis of acromegaly. They have also shown that CTS surgery was performed prior to the diagnosis of acromegaly in 5-17% of patients [6]. In our patient, the diagnosis of acromegaly was made four years after the beginning of symptoms of CTS, five months after its confirmation by electrophysiological study, and two months after surgical decompression. Concerning electrophysiological analysis and imaging in CTS, cohorts of acromegalic patients indicate a delay of nerve conduction with variable frequencies (42% - 81%) and enlarged median nerves [5,7,8].

In our case, the electrophysiological analysis showed a bilateral delay of the distal motor latencies (35.4 ms) of the median nerves with a reduction in the amplitudes of their motor and sensory responses and a marked reduction in their sensory conduction velocities. Finally, the control of acromegaly improves the symptoms of CTS according to several studies. Sasagawa *et al*. [5] found that CTS symptoms disappeared after controlling GH hypersecretion by surgery alone or by surgery and adjuvant medical treatment.

Furthermore, Tagliafico *et al*. [7] examined the median nerve and ulnar nerve of 34 acromegalic patients with ultrasound. They noticed that median and ulnar nerves presented a greater cross-sectional area in the case of uncontrolled disease compared to the control one. They suggested that the enlargement peripheral nerve may be an intrinsic feature of the disease. In our case, the patient reports improvement in CTS symptoms after median nerve decompression surgery and is scheduled for surgery on pituitary adenoma.

## Conclusion

Acromegaly is a serious disease because of its complications. The guidelines emphasize the importance of its early diagnosis. Carpal tunnel syndrome is frequent in acromegaly and it is often the initial complaint. Remember to look for other signs of acromegaly in front of the CTS might help to make an early diagnosis of acromegaly.
